# Case Report: Unmasked Inherited Dysfibrinogenemia After Everolimus Therapy

**DOI:** 10.3389/fmed.2020.591546

**Published:** 2020-11-27

**Authors:** Alona A. Merkulova, Steven C. Mitchell, Sergei Merkulov, Alisa S. Wolberg, Marguerite Neerman-Arbez, Alvin H. Schmaier

**Affiliations:** ^1^Department of Medicine, Hematology and Oncology, Case Western Reserve University, Cleveland, OH, United States; ^2^Department of Medicine, Cardiovascular Medicine, Case Western Reserve University, Cleveland, OH, United States; ^3^Department of Pathology and Laboratory Medicine, University of North Carolina, Chapel Hill, NC, United States; ^4^Department of Genetic Medicine and Development, University Medical Center Geneva, Geneva, Switzerland; ^5^Division of Angiology and Haemostasis, University Hospital, Geneva, Switzerland; ^6^Department of Medicine, Hematology and Oncology, University Hospitals Cleveland Medical Center, Cleveland, OH, United States

**Keywords:** dysfibrinogenemia, everolimus, CVID, fibrin polymerization, dysfibrinogenemia Krakow III

## Abstract

A previously hemostatically asymptomatic patient with common variable hypogammaglobulinemia was given everolimus to prevent growth of her liver. Within several months, the patient developed a severe bleeding disorder. The bleeding was due to fibrin polymerization defect that upon sequencing was shown to be dysfibrinogenemia Krakow III. Elimination of the mTor inhibitor ameliorated the clinical bleeding state.

A 45 yo woman with a history of common variable immunodeficiency (CVID) was referred for a bleeding disorder. In 2009, this patient was diagnosed with common variable hypogammaglobulinemia. In 2011, the disorder manifested with immune thrombocytopenia and a splenectomy was performed. At that time, she had a normal prothrombin time (PT) and activated partial thromboplastin time (aPTT) and had no abnormal bleeding at surgery. In 2013, she developed hepatomegaly. In 2016, the hepatomegaly was symptomatic and she was started on everolimus by her liver immunologists as part of a protocol to reduce her liver size.

Serious, spontaneous bleeding started 3–6 months after starting everolimus. It began with recurrent lower GI hemorrhages. Since 2016, this patient has had multiple hospitalizations for spontaneous GI bleedings, intra-abdominal hemorrhages, and hematomas in the right sacral plexus, right ankle, and multiple soft tissue locations. The sacral plexus bleed gave the patient a persistent right foot drop. On presentation at our hospital, the patient had a slightly prolonged aPTT [43 ± 6.6 (Mean ± SD) (normal 28–38 s)] but a normal PT [11.4 ± 0.7 (normal 9.7–12.7s)] (Figure 1A). Blood coagulation factors XII (1.41 U/ml), prekallikrein (1.38 U/ml), high molecular weight kininogen (0.85 U/ml), XI (0.63–0.79 U/ml), IX (0.79–1.17 U/ml), VIII (1.27 U/ml), VII (0.68–1.02 U/ml), X (0.69–0.89 U/ml), V (0.95 U/ml), and II (0.65–0.99 U/ml) were always normal. The reported range of values for factors XI, IX, VII, X, and II are from multiple assays over a 3-years period.

**Figure 1:**
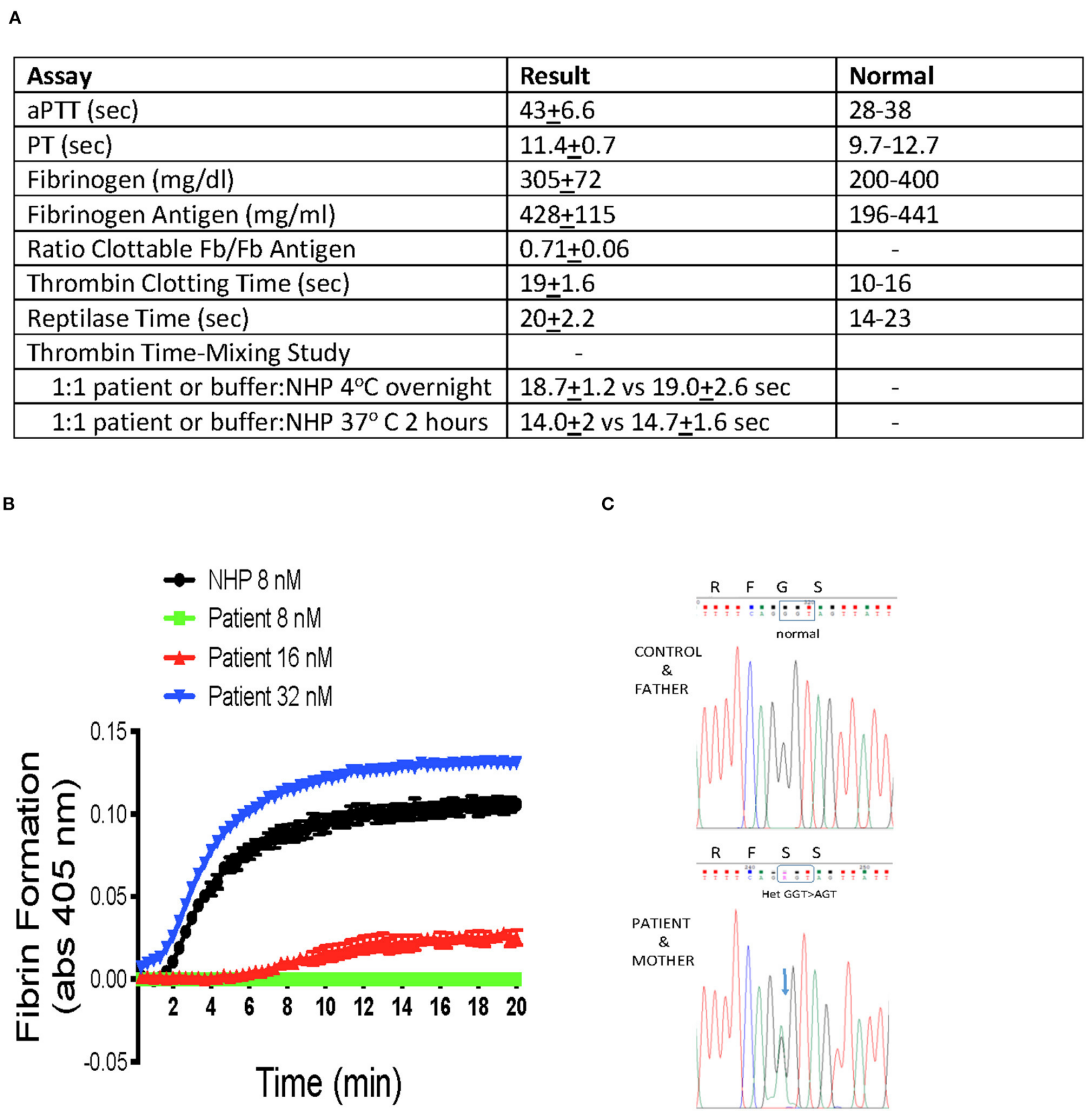
**(A)** Summary of coagulation studies. Patient samples were collected in 3.2% sodium citrate (1:9 anticoagulant to total blood volume ratio). The aPTT, PT, clottable fibrinogen, fibrinogen antigen, thrombin clotting time, and reptilase time were performed in the Clinical Laboratories of University Hospital Cleveland Medical Center, Cleveland, OH. Thrombin time mixing studies were performed by a 1:1 incubation of patient or normal human plasma (NHP) with NHP or buffer, respectively, and incubated: one set was incubated overnight 16 h at 4°C, and a second set was incubated 2 h at 37°C. At the conclusion of the incubation, the samples received an equal volume of 8 NIH Units/ml of human α-thrombin (Enzyme Research Laboratories 3,000 U/mg specific activity) in 30 mM calcium chloride and the time to clot formation was recorded with a stop watch. The final thrombin concentration was 4 NIH U/ml or 0.5 nM α -thrombin. **(B)** Fibrin polymerization studies. Patient or NHP were added to 96-well microtiter plates. Upon the addition of buffer containing 8–32 nM human α-thrombin and 30 mM CaCl_2_, continuous visible light recording for turbidity at 405 nm were performed every 20 s for 20 min. The figure shows the mean ± SD of progress curves of three samples of patient or normal plasma treated with the indicated concentration of α-thrombin. **(C)** Family Genetic Studies. The figure is the sequence of the glycine 16 region of human γ-chain of fibrinogen from DNA isolated from buffy coat of Control (normal donor), Patient, and Patient's Mother and Father. Sequence was determined by PCR amplification followed by Sanger sequencing.

The patient's clottable fibrinogen [305 ± 72 mg/dL (normal 200–400 mg/dl)] and fibrinogen antigen [428 ± 115 mg/dL (normal 196–441)] were both normal (Figure 1A). However, the clottable fibrinogen-to-fibrinogen antigen ratio was low (0.71 ± 0.06). The patient's reptilase time was normal [20 ± 2.2 (normal 14–23 s)], but her thrombin time was persistently prolonged [19 ± 1.6 (normal 10–16 s)]. On mixing test of patient plasma 1:1 with normal plasma for 2 h at 37°C or overnight at 4°C, there was nothing in the patient plasma that prolonged the thrombin clotting of normal human plasma (Figure 1A). The patient had slightly elevated alpha-1-antitrypin, [231 mg/dl (normal 84–218)], but normal antithrombin activity [105% (normal 80–130)] and antigen [91 (normal 80–120)].

In general, a normal reptilase time with an abnormal thrombin time suggests a fibrinopeptide B release defect (1, 2). However, fibrinopeptide B defects are not associated with bleeding (2). Therefore the combined clinical and laboratory data suggest a fibrin polymerization defect. In a fibrin polymerization assay, the patient required 4-fold greater concentrations of human alpha-thrombin (32 vs. 8 nM) to achieve complete fibrin polymerization (Figure 1B). Since recognizing this fibrin polymerization defect, the patient's bleeding episodes have been controlled by cryoprecipitate infusions to raise her baseline fibrinogen values by 150–250 mg/dl with normal fibrinogen.

Sequencing of fibrinogen coding regions following PCR amplification of leukocyte genomic DNA revealed that the patient was heterozygous for a mutation in fibrinogen gamma chain exon 3 [*FGGc*.124G>A, p.Gly42Ser (Gly16Ser in the mature protein without the signal peptide)] (Figure 1C). This defect was previously described in a bleeding patient with similar blood coagulation studies as Fibrinogen Krakow III (3). Neither a control DNA sample from a normal donor nor the patient's father have this mutation. However, the patient's mother has the identical heterozygous mutation. The patient's mother, who is otherwise healthy, has no bleeding history. Our patient also is heterozygous for Fibrinogen Krakow III but never had a bleeding problem until everolimus treatment was instituted to manage her CVID.

The original hypothesis for these investigations was that treatment with everolimus caused an “acquired” bleeding state. Everolimus is an mTor inhibitor and mTor is a major regulator of protein synthesis (4). Further, mTor inhibition itself blocks fibrin clot retraction (5). Later, the patient also was found to have a heterozygous genetic polymorphism in her fibrinogen's gamma chain that interferes with fibrin polymerization. Thus, the patient's congenital fibrinogen mutation and everolimus treatment combined to give this patient a serious bleeding disorder. Upon urging the patient and treating physicians, the everolimus therapy was stopped and she has not had a major bleeding incident in 18 months. She does, however, have frequent minor mucous membrane bleeding (e.g., epistaxis). She persists in having an abnormal thrombin time and reduced fibrinogen activity/antigen ratio of 0.7.

## Data Availability Statement

The authors acknowledge that the data presented in this study must be deposited and made publicly available in an acceptable repository, prior to publication. Frontiers cannot accept a article that does not adhere to our open data policies.

## Ethics Statement

The studies involving human participants were reviewed and approved by University Hospitals Cleveland Medical Center IRB. The patients/participants provided their written informed consent to participate in this study. Written informed consent was obtained from the individual(s) for the publication of any potentially identifiable images or data included in this article.

## Author Contributions

AM, SMi, SMe, AW, and MN-A contributed experimental studies. AS conceived the project. AS, AW, and MN-A wrote the manuscript. All authors contributed to the article and approved the submitted version.

## Conflict of Interest

The authors declare that the research was conducted in the absence of any commercial or financial relationships that could be construed as a potential conflict of interest.
